# Structure‐activity relationship study of neuroprotective complex I inhibitor CP2

**DOI:** 10.1002/alz.085971

**Published:** 2025-01-09

**Authors:** Olga Petrova, Sergey A Trushin, Thi Kim Oanh Nguyen, Mark Ostroot, Matthew Schellenberg, Graham Johnson, Eugenia Trushina, Leonid Sazanov

**Affiliations:** ^1^ Institute of Science and Technology Austria (ISTA), Klosterneuburg Austria; ^2^ Department of Neurology, Mayo Clinic, Rochester, MN USA; ^3^ Mayo Clinic, Rochester, MN USA; ^4^ NuPharmAdvise LLC, Sanbornton, NH USA; ^5^ Department of Molecular Pharmacology and Experimental Therapeutics, Mayo Clinic, Rochester, MN USA

## Abstract

**Background:**

We identified small molecule tricyclic pyrone compound CP2 as a mild mitochondrial complex I (MCI) inhibitor that induces neuroprotection in multiple mouse models of AD. One of the major concerns while targeting mitochondria is the production of reactive oxygen species (ROS). CP2 consists of two diastereoisomers, D1 and D2, with distinct activity and toxicity profiles. This study was designed to understand how structure of D1 and D2 affects their binding to MCI and the consequential impact on ROS production.

**Method:**

The X‐ray crystallography and cryo‐electron microscopy (cryo‐EM) at global resolution of 3.25‐3.27Å were employed to identify the molecular structure of D1 and D2 and the D1 binding to the isolated ovine MCI. The assessment of the MCI inhibition and the extent of ROS generation were done in isolated MCI and human neuroblastoma MC65 cells using flow cytometry, a Seahorse extracellular flux analyzer, and the kinetic studies.

**Result:**

In the closed conformation of MCI, D1 selectively binds to the deep Quinone‐site (Q_d_) but not to the shallow Q‐site (Q_s_), sharing the same binding pocket as rotenone. In the open MCI state, D1 exclusively binds to the Q_s_ in contrast to rotenone, which binds Q_d_ and Q_s_ in both closed and open states. At the same concentrations, D1 inhibits respiration to a greater extent compared to D2 (5:1 ratio) and produces higher level of ROS.

**Conclusion:**

Cryo‐EM unambiguously identified binding of D1 to both the Q_d_ and Q_s_ sites, contingent upon the conformational state of MCI. In contrast to rotenone, D1 binds Q_d_ only in the closed conformation during catalytic cycle, leading to mild inhibition. Superimposing X‐ray crystallography data of D1 and D2 onto cryo‐EM data suggests that the orientation of the methyl group in D2 induces a flatter conformation, resulting in lower binding affinity to MCI, which correlates with lower inhibition and toxicity compared to D1. At physiologically relevant concentrations, CP2 (D1:D2 = 1:1) demonstrates low MCI inhibition yielding negligible ROS levels. This observation provides new insight into the absence of toxicity associated with CP2 treatment *in vivo*, further highlighting feasibility for the development of safe and efficacious MCI inhibitors.

